# Epidemiology, risk factors and outcomes of bloodstream infection caused by ESKAPEEc pathogens among hospitalized children

**DOI:** 10.1186/s12887-021-02661-9

**Published:** 2021-04-21

**Authors:** Xiaoshan Peng, Wei Zhou, Yu Zhu, Chaomin Wan

**Affiliations:** 1 Department of Pediatrics, West China Second Hospital, Sichuan University, No 20, 3rd section of Renmin South Road, 610041 Chengdu, China; 2 Key Laboratory of Birth Defects and Related Diseases of Women and Children (Sichuan University), Ministry of Education, Chengdu, China; 3 Department of Clinical Microbiology Laboratory, West China Second Hospital, Sichuan University, Chengdu, China

**Keywords:** ESKAPEEc pathogens, Bloodstream infection, Children

## Abstract

**Background:**

Bloodstream infection (BSI) resulting from ESKAPEEc pathogens (*Enterococcus faecium*, *Staphylococcus aureus*, *Klebsiella pneumoniae*, *Escherichia coli*, *Acinetobacter baumannii*, *Pseudomonas aeruginosa* and Enterobacter spp) is relevant to high mortality and economic cost. Data concerning the impact of BSI due to ESKAPEEc in pediatric population was virtually scant. Our purpose was to summarize the epidemiology, risk factors and outcomes of ESKAPEEc BSI among hospitalized children.

**Methods:**

Inpatients diagnosed with BSI with definite etiology between January 2016 and December 2018 were enrolled retrospectively at the West China Second University Hospital. Data were systematically reviewed on patients’ clinical characteristics and laboratory findings to ascertain independent predictors, clinical features and outcomes.

**Results:**

Of the 228 patients with BSI, 174 (76.3%) were caused by ESKAPEEc (124 MDR-ESKAPEEc). Multivariate analysis demonstrated that premature and/ or low birth weight (odds ratio [OR] = 2.981, *P* = 0.036), previous surgery and/or trauma (OR = 5.71, *P* = 0.029) and source of urinary tract infection (OR = 10.60, *P* = 0.004) were independently associated with ESKAPEEc BSI. The independent risk factor for MRD-ESKAPEEc BSI was nosocomial infection (OR = 3.314, *P* = 0.037). The overall mortality rate in patients with ESKAPEEc BSI was 14.4% (25/174), and no significant difference was ascertained in mortality between MRD-ESKAPEEc and non-MRD ESKAPEEc BSI groups (13.7% vs. 11.4%, *P* = 0.692). In addition, previous surgery and/or trauma, thrombocytopenia, and mechanical ventilation were significant risk factors for mortality caused by ESKAPEEc BSI.

**Conclusions:**

More than two-thirds of BSI among hospitalized children were caused by ESKAPEEc. Previous surgery and/or trauma, thrombocytopenia and mechanical ventilation increased the risk rate for mortality in ESKAPEEc BSI. The risk factors ascertained could assist physicians to early suspect ESKAPEEc BSI and MDR ESKAPEEc BSI.

## Background

The notorious group of pathogens, *Enterococcus faecium*, *Staphylococcus aureus*, *Klebsiella pneumoniae*, *Escherichia coli*, *Acinetobacter baumannii*, *Pseudomonas aeruginosa* and Enterobacter spp., named “ESKAPEEc” owing to their high resistances to multiple antimicrobial agents, have recently aroused global concern [1, 2]. Currently, the incidence of bloodstream infection (BSI) caused by ESKAPEEc has increased rapidly [3], and ESKAPEEc BSI brought about worse outcomes [3], longer hospital stays, higher economic costs [4–6], and increased mortality [4–6]. In addition, effective antimicrobial agents against ESKAPEEc strains were limited due to the growth of resistance to multiple antibiotics in these bacterial species. Inappropriate and delayed empirical antimicrobial agents’ treatment for patients with BSI was connected with high risk of mortality [7–10]. Therefore, a wide understanding of the main clinical characteristics of ESKAPEEc BSI among hospitalized children was crucial for physicians to early recognition and select proper empirical therapy.

To our knowledge, the existing studies have given attention to the epidemiology and antimicrobial resistance trends of ESKAPEEc in patients with BSI [11–13], and clinical data about ESKAPEEc BSI were mainly available in adult populations with cancer or solid organ transplantation [14–17]. So far, there was no data concerning the BSI caused by ESKAPEEc in pediatric populations. Thus, we sought to investigate the epidemiology, clinical characteristics among hospitalized children with ESKAPEEc BSI. Simultaneously, we also assessed risk factors, and clinical outcomes of them.

## Methods

### Study population

**Fig. 1:**
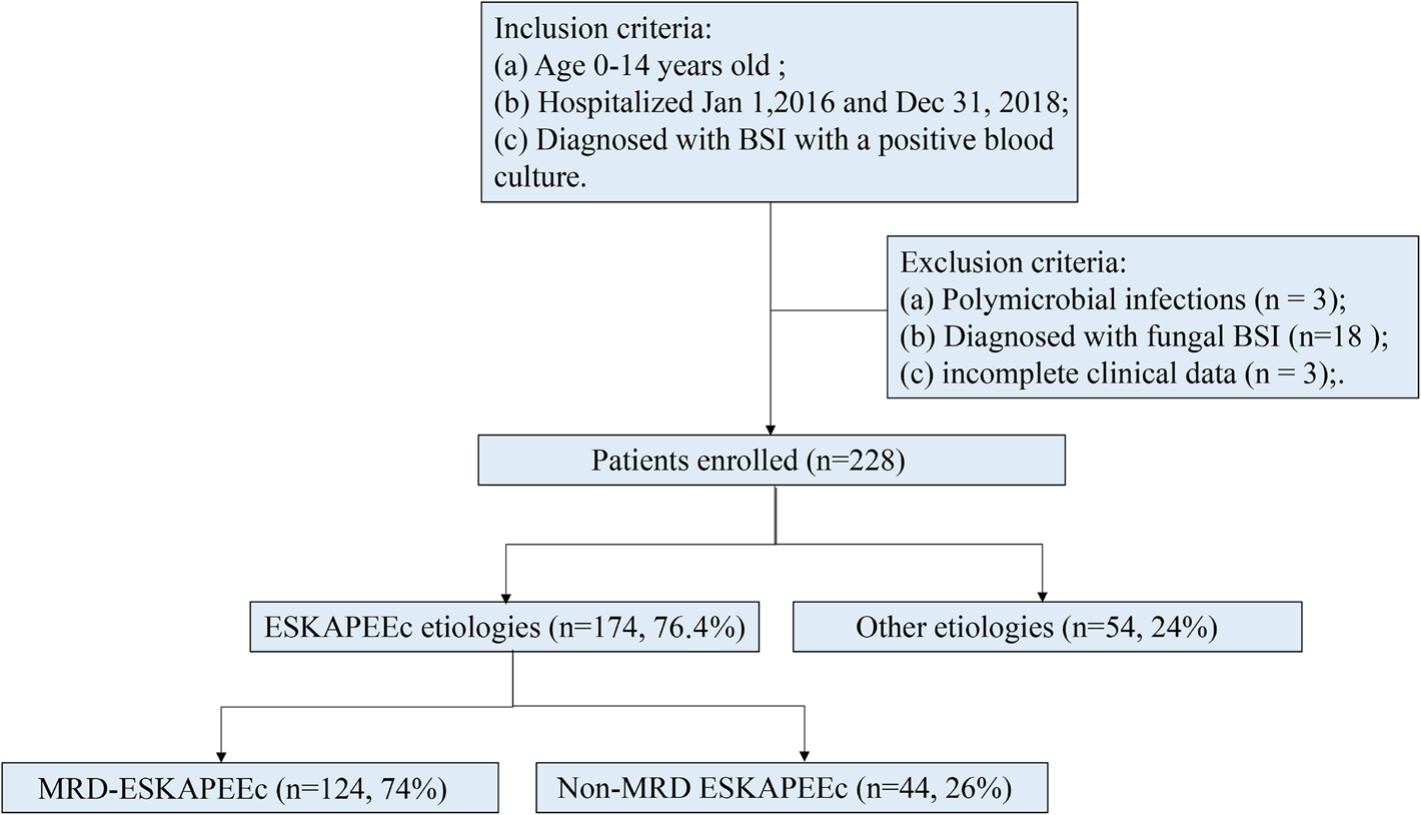
Flow diagram for the study patients. BSI, bloodstream infection; MDR, multidrug resistance

Hospitalized children who were diagnosed with BSI based on a positive blood culture between 2016 and 2018 at the West China Second University Hospital of Sichuan University and aged under 14 years old were enrolled retrospectively after obtaining ethics approval. The exclusion criteria were listed next (Fig. 1): (1) polymicrobial infections;(2) diagnosed with fungal BSI; (3) incomplete clinical data.

### Data collection

This study obtained the following data from electronic medical records, including age, gender, previous hospitalization (within 1 month), history of surgery and/ or trauma (within 3 months), records of previous antibiotic use (within 1 month), underlying diseases, nosocomial infection or not, symptoms, microbiology data (microorganisms and resistance to antimicrobial agents), likely source of infection, blood products transfusion, pediatric intensive care unit (PICU) admission, invasive operation (indwelling gastric tube, central venous catheter, urinary catheter, mechanical ventilation), empirical antibiotics therapy, length of hospital stay and patients’ clinical outcomes. Meanwhile, the additional laboratory results within the first 24 h of admission were collected: the blood routine, C-reactive protein, alanine aminotransferase (ALT) and aspartate aminotransferase (AST). This study was analyzed through three parts. First, all enrolled patients were classified into ESKAPEEc and non-ESKAPEEc groups to ascertain the risk factors of ESKAPEEc BSI. Second, risk factors of patients with BSI resulting from MRD ESKAPEEc were investigated by comparing the MRD ESKAPEEc and non-MRD ESKAPEEc groups. Finally, the 174 patients with ESKAPEEc BSI according to patients’ final condition at discharge were divided into survivor and non-survivor groups to investigate the risk factors closely connected with mortality.

### Definitions

BSI was defined as the causative bacteria isolated in the blood cultures and clinical manifestations in accordance with sepsis syndrome [18]. The diagnosis criteria were determined by the definitions of National Healthcare Safety Network and CDCP [19]. ESKAPEEc were previously defined elsewhere [1, 2, 4, 12]. The definition of MRD was based on the international expert proposal for interim standard [20]. Polymicrobial infection referred to ≥2 causative organisms isolated from a single blood culture [21]. Empirical antibiotic treatment was regarded as being “appropriate” if the isolated causative organisms were susceptible to ≥1 of the antimicrobial agents administered in vitro. Otherwise, the treatment was considered to be “inappropriate”. Overall mortality referred to death by any cause during hospitalization. Some children who had multiple organ failure, demanded inotropes support or mechanical ventilation were discharged from hospital voluntarily and gave up any further treatment. These children were considered non-survivor groups within this period.

### Microbiological Methods

The blood cultures were fulfilled strictly in accordance with the standard operating procedures. The causative bacteria identification and antibiotic resistance testing were conducted using the VITEK 2 (BioMérieux, Marcy-l’Étoile, France) system. The interpretation criteria of antibiotic resistance tests was determined by the definitions of Clinical and Laboratory Standards Institute [22].

### Statistical analysis

We reported the median (interquartile range [IQR]) for measurement dates and analyzed them using Mann-Whitney U test appropriately. We calculated constituent ratios for count dates and analyzed them utilizing the χ^2^ test or Fisher’s exact test. Variables in the univariate analysis that exhibited statistical differences (*P*-value < 0.05) were placed into the binary logistic regression analysis to investigate factors potentially associated with ESKAPEEc BSI, MRD-ESKAPEEc BSI and mortality. All dates were implemented with the SPSS version 22.0. *P*-values < 0.05 were deemed significant.

## Results

Two hundred fifty-two hospitalized pediatric patients were diagnosed with laboratory-confirmed BSI Between 2016 and 2018 at the West China Second Hospital, Sichuan University. Twenty-four patients were not included according to the exclusion criteria (Fig. 1); Ultimately, we identified 228 patients in this study. One hundred seventy-four of the 228 patients (76.3%) had ESKAPEEc BSI. Six of the 174 (3.4%) patients lacked resistance data; 124 (73.8%) were MDR ESKAPEEc and 44 (26.2%) were non-MDR ESKAPEEc.

### Microbiology

As Table 1 showed, the two leading ESKAPEEc pathogens were *Escherichia coli* (26.8%,61/228), *Klebsiella pneumoniae* (20.2%,46/228), followed by *Enterococcus faecium* (12.7%, 29/228), *Staphylococcus aureus* (12.7%, 29/228), *Acinetobacter baumannii* (2.6%, 6/228), *Pseudomonas aeruginosa* (1.3%, 3/228) and Enterobacter spp. (0). Of the 124 MDR ESKAPEEc strains, *Escherichia coli* and *Klebsiella pneumoniae* accounted for nearly 65.3%. 42 (33.9%) were ESBL-producing bacteria, including *Escherichia coli* (28,22.6%) and *Klebsiella pneumoniae* (14,11.3%). 20 (16.1%) were carbapenem non-susceptibility bacteria, including *Klebsiella pneumoniae* (15,12.1%), *Escherichia coli* (3,2.4%) and *Acinetobacter baumannii* (2,1.6%). In addition, there were 1 vancomycin-resistant *Enterococcus faecium* and 8 methicillin-resistant *Staphylococcus aureus*.

**Table 1:** Bacteria isolated in hospitalized children with bloodstream infection

Organism	*N* (%)	MDR ESKAPEEc, *N*(%)
*Enterococcus faecium*	29 (12.7)	23 (18.5)
*Staphylococcus aureus*	29 (12.7)	12 (9.7)
*Klebsiella pneumoniae*	46 (20.2)	34 (27.4)
*Pseudomonas aeruginosa*	3 (1.3)	3 (2.4)
*Acinetobacter baumannii*	6 (2.6)	5 (4.0)
Enterobacter spp	0	0
*Escherichia coli*	61 (26.8)	47 (37.9)
*Streptococcus pneumoniae*	27 (11.8)	
Group B Streptococcus	9 (3.9)	
*Enterococcus faecalis*	5 (2.2)	
Salmonella spp	13 (5.7)	
Total	228 (100)	124 (100)

*Abbreviations*: *MDR* multidrug resistance

### Comparison of ESKAPEEc and non-ESKAPEEc BSI

The main characteristics of patients with ESKAPEEc and non-ESKAPEEc BSI were summarized in Table 2. Less than half of the patients were males (42.1%, 96/228) and the median age was 4.4 (range:0.7–35.7) months. The median age between the two groups (median month, 2.5 [0.5–12.3] vs 32.7[8.0–100.4], *P* < 0.001) showed statistically significant difference. Regarding underlying disease, a greater proportion of premature and/or low birth weight (31.6% vs 9.3%, *P* = 0.001), tumor diseases (16.7% vs 0%, *P* < 0.001) was presented in patients with ESKAPEEc BSI. Compared with non-ESKAPEEc BSI, patients with BSI caused by ESKAPEEc had increased percentages of previous surgery and/ or trauma (13.8% vs 3.7%, *P* = 0.042), nosocomial infection (32.2% vs 7.4%, *P* < 0.001), more source of urinary tract infection (14.9% vs 1.9%, *P* = 0.009), but lower rate of previous antibiotic use (25.9% vs 50.0%, *P* = 0.001), less source of lung infection (17.8% vs 42.6%, *P* < 0.001), intracranial infection (1.1% vs 16.7%, P < 0.001) and lower levels of platelet count (median, 209 [111–323] vs 341 [168–439], P < 0.001), C-reactive protein (median mg/dL,15.0 [3.0–85.3] vs 26.5 [10.0–116.6], *p* = 0.017). After applying the multivariate analysis, premature and/or low birth weight (odds ratio [OR] = 2.981, *P* = 0.036), previous surgery and/or trauma (OR = 5.71, *P* = 0.029) and source of urinary tract infection (OR = 10.6, *P* = 0.004) were independent risk factors for ESKAPEEc BSI. Source of intracranial infection was a protective factor for ESKAPEEc BSI (OR = 0.198, *P* = 0.037).

**Table 2:** Comparison of ESKAPEEc and non-ESKAPEEc bloodstream infections among hospitalized children

Variable	ESKAPEEC *n* = 174	Non-ESKAPEEC *n* = 54	Univariate analysis	Logistic Regression Analysis^#^	
			*P* Value	*P* Value	Odds Ratio (95% CI)
Male, No. (%)	71 (40.8)	25 (46.3)	0.475		
Age (m), Median (IQR)	2.5 (0.5–12.3)	32.7 (8–100.4)	< 0.001	0.367	1.004 (0.995–1.014)
Presence of any underlying disease, No. (%)	111 (63.8)	14 (25.9)	< 0.001		
Premature and/or low birth weight, No. (%)	55 (31.6)	5 (9.3)	0.001	0.036	2.981 (1.076–8.257)
Tumor diseases, No. (%)	29 (16.7)	0	< 0.001	0.262	2.476 (0.508–12.077)
Aplastic anemia, No. (%)	3 (1.7)	2 (3.7)	0.737		
Congenital heart disease, No. (%)	11 (6.3)	1 (1.9)	0.349		
Lung disease, No. (%)	12 (6.9)	1 (1.9)	0.289		
Digestive disease, No. (%)	15 (8.6)	1 (1.9)	0.163		
Kidney disease, No. (%)	4 (2.3)	1 (1.9)	1.000		
Other diseases, No. (%)	11 (6.3)	3 (5.6)	1.000		
Nosocomial infection, No. (%)	56 (32.2)	4 (7.4)	< 0.001	0.579	1.39 (0.435–4.447)
Previous surgery and/ or trauma (within 3 months), No. (%)	24 (13.8)	2 (3.7)	0.042	0.029	5.71 (1.191–27.384)
Previous hospitalization (within 1 month), No. (%)	33 (19.0)	7 (13.0)	0.311		
Previous antibiotic use (within 1 month), No. (%)	45 (25.9)	27 (50.0)	0.001		
Penicillins, No. (%)	8 (4.6)	7 (13.0)	0.064		
Cephalosporins, No. (%)	21 (12.1)	18 (33.3)	< 0.001	0.168	0.558 (0.243–1.279)
β-lactam-β-lactamase inhibitor combination regimens, No. (%)	18 (10.3)	3 (5.6)	0.427		
Carbapenems, No. (%)	6 (3.4)	2 (3.7)	1.000		
Macrolides, No. (%)	1 (0.6)	3 (5.6)	0.065		
Glycopeptides, No. (%)	8 (4.6)	0	0.238		
Others, No. (%)	1 (0.6)	3 (5.6)	0.065		
Likely source of infections					
Lung infection, No. (%)	31 (17.8)	23 (42.6)	< 0.001	0.098	0.533 (0.254–1.122)
Abdominal infection, No. (%)	27 (15.5)	5 (9.3)	0.247		
Urinary infection, No. (%)	26 (14.9)	1 (1.9)	0.009	0.004	10.6 (2.118–53.044)
Intracranial infection, No. (%)	2 (1.1)	9 (16.7)	< 0.001	0.037	0.198 (0.043–0.906)
Skin or soft tissue infection, No. (%)	20 (11.5)	1 (1.9)	0.061		
Primary BSI, No. (%)	75 (43.1)	16 (29.6)	0.077		
Laboratory findings					
White blood cell count (*10^9),Median (IQR)	9.4 (5.0–13.7)	11.1 (6.4–17.0)	0.106		
Neutrophil percentage, Median (IQR)	56.0 (33.9–71.8)	56.9 (37.4–76.5)	0.370		
Hemoglobin (g/L), Median (IQR)	108.0 (93.0–144.5)	106.0 (90.3–125.0)	0.272		
Platelet count ((*10^9), Median (IQR)	209 (111–323)	341 (168–439)	< 0.001	0.142	0.999 (0.997–1.000)
C-reactive protein (mg/dL), Median (IQR)	15.0 (3.0–85.3)	26.5 (10.0–116.6)	0.017	0.704	1.001 (0.995–1.007)
Elevated ALT, No. (%)	56 (32.2)	20 (37.0)	0.509		
Elevated AST, No. (%)	90 (51.7)	21 (38.9)	0.099		
Severity of BSI					
MODS, No. (%)	15 (8.6)	6 (11.1)	0.777		
Septic shock, No. (%)	13 (7.5)	2 (3.7)	0.508		
Mechanical ventilation, No. (%)	44 (25.3)	13 (24.1)	0.857		
PICU admission, No. (%)	31 (17.8)	9 (16.7)	0.846		
Length of hospital stay (days), Median (IQR)	20.5 (10.0–31.0)	14.0 (8.8–23.0)	0.023		
7-day mortality	14 (8.0)	4 (7.4)	0.879		
Overall mortality	25 (14.4)	7 (13.0)	0.795		
Inappropriate empirical treatment, No. (%)	46 (27.4)*	4 (7.4)	0.003		

*Abbreviations*: *ALT* alanine aminotransferase, *AST* aspartate aminotransferase, *BSI* bloodstream infection, *MODS* multiple organ dysfunction syndrome, *IQR* interquartile range, *CI* confidence interval, *PICU* pediatric intensive care unit

Elevated ALT means ALT was > 40 U/L. Elevated AST means AST was > 45 U/L.

^#^ *P* value of Hosmer-Lemeshow goodness-of-fit test was 0.181

* Percentage derived  from the result of ESKAPEEC BSI patients with resistance data (*n*=168)

In terms of empiric antimicrobial treatment and outcomes of all cases, we found that 50 of the 228 patients (21.9%) were treated inappropriately: 46 (27.4%, 46/168) were in ESKAPEEc BSI patient group and 4 (7.4%,4/54) were in non-ESKAPEEc BSI group. Patients with ESKAPEEc BSI had received inappropriate empiric antibiotics treatment significantly more often (27.4% vs 7.4%, *p* = 0.003) (Table 2). Although no significant differences in the MODS, septic shock, mechanical ventilation, PICU admission and mortality between ESKAPEEc and non-ESKAPEEc BSI groups were found (all *p* > 0.05). Patients with BSI due to ESKAPEEc had longer hospital stay (median days, 20.5[10.0–31.0] vs 14.0[8.8–23.0], *p* = 0.023) compared with those with non-ESKAPEEc BSI.

### Comparison of MDR ESKAPEEc and non-MDR ESKAPEEc BSI

The 168 patients with resistance data were divided into MDR ESKAPEEc and non-MDR ESKAPEEc BSI groups. The differences of the main characteristics between the 2 groups were showed in Table 3. MDR ESKAPEEc patients with BSI had more nosocomial infections (41.1% vs 11.4%, *P* < 0.001) and the presence of underlying disease (72.6% vs 43.2%, *P* < 0.001) was also higher, whereas the age and sex between the 2 groups were not statistically different. Compared with non-MDR ESKAPEEc BSI, patients with MDR-ESKAPEEc BSI were less likely to have a history of antibiotic use within 1 month (21.8% vs 43.2%, *P* = 0.006), source of skin or soft tissue infection (6.5% vs 27.3%, *P* < 0.001). Furthermore, the median level of platelet count was significantly lower in MDR-ESKAPEEc group with BSI than that in non-MDR ESKAPEEc group (median,188[100–302] vs 271[173–413], *P* = 0.004). In multivariate analysis, the independent risk factor for MRD-ESKAPEEc BSI was nosocomial infection (OR = 3.314, *P* = 0.037), while the skin or soft tissue infection (OR = 0.245, *P* = 0.011) was a protective predictor of MRD ESKAPEEc BSI.

**Table 3:** Comparison of MRD and non-MRD ESKAPEEc bloodstream infections among hospitalized children

Variable	MRD-ESKAPEEC *n* = 124	Non-MRD ESKAPEEC *n* = 44	Univariate analysis	Logistic Regression Analysis^#^	
			*P* Value	*P* Value	Odds Ratio (95% CI)
Male, No. (%)	50 (40.3)	18 (40.9)	0.946		
Age (m), Median (IQR)	2.5 (0.5–26.4)	9.7 (1.0–72.0)	0.07		
Presence of any underlying disease, No. (%)	90 (72.6)	19 (43.2)	< 0.001	0.469	1.376 (0.581–3.26)
Premature and/or low birth weight, No. (%)	44 (35.5)	10 (22.7)	0.120		
Tumor diseases, No. (%)	24 (19.4)	5 (11.4)	0.228		
Aplastic anemia, No. (%)	3 (2.4)	0	0.568		
Congenital heart disease, No. (%)	10 (8.1)	1 (2.3)	0.327		
Lung disease, No. (%)	11 (8.9)	1 (2.3)	0.263		
Digestive disease, No. (%)	14 (11.3)	1 (2.3)	0.135		
Kidney disease, No. (%)	3 (2.4)	1 (2.3)	1.000		
Other diseases, No. (%)	7 (5.6)	3 (6.8)	1.000		
Nosocomial infection, No. (%)	51 (41.1)	5 (11.4)	< 0.001	0.037	3.314 (1.076–10.205)
Previous surgery and/ or trauma (within 3 months), No. (%)	20 (16.1)	4 (9.1)	0.252		
Previous hospitalization (within1 month), No. (%)	27 (21.8)	6 (13.6)	0.243		
Previous antibiotic use (within 1 month), No. (%)	27 (21.8)	19 (43.2)	0.006	0.219	0.6 (0.266–1.356)
Likely source of infections					
Lung infection, No. (%)	27 (21.8)	4 (9.1)	0.062		
Abdominal infection, No. (%)	20 (16.1)	7 (15.9)	0.973		
Urinary infection, No. (%)	17 (13.7)	9 (20.5)	0.288		
Intracranial infection, No. (%)	2 (1.6)	0	1.000		
Skin or soft tissue infection, No. (%)	8 (6.5)	12 (27.3)	< 0.001	0.011	0.245 (0.083–0.721)
Primary BSI, No. (%)	55 (44.4)	14 (31.8)	0.146		
Laboratory findings					
White blood cell count (*10^9),Median (IQR)	9.4 (4.9–13.9)	9.8 (7.0–13.3)	0.358		
Neutrophil percentage, Median (IQR)	54.0 (33.4–71.0)	58.8 (41.0–76.9)	0.082		
Hemoglobin (g/L), Median (IQR)	110.0 (91.8–150.0)	104.5 (94.0–126.0)	0.424		
Platelet count ((*10^9), Median (IQR)	188 (100–302)	271 (173–413)	0.004	0.076	0.998 (0.996–1.000)
C-reactive protein (mg/dL), Median (IQR)	9.5 (3.0–65.8)	32.5 (2.3–150.7)	0.203		
Elevated ALT, No. (%)	39 (31.5)	13 (29.5)	0.814		
Elevated AST, No. (%)	68 (54.8)	18 (40.9)	0.112		
Severity of BSI					
MODS, No. (%)	12 (9.7)	1 (2.3)	0.211		
Septic shock, No. (%)	10 (8.1)	0	0.065		
Mechanical ventilation, No. (%)	32 (25.8)	10 (22.7)	0.685		
PICU admission, No. (%)	18 (14.5)	11 (25.0)	0.114		
Length of hospital stay (days), Median (IQR)	24.0 (13.0–36.0)	14.5 (9.0–27.3)	0.006		
7-day mortality	8 (6.5)	3 (6.8)	1.000		
Overall mortality	17 (13.7)	5 (11.4)	0.692		
Inappropriate empirical treatment, No. (%)	42 (33.9)	4 (9.1)	0.002		

*MDR* multidrug resistant. The interpretation for the other abbreviations were listed in Table 2 legend

Elevated ALT means ALT was > 40 U/L. Elevated AST means AST was > 45 U/L.

^#^ *P* value of Hosmer-Lemeshow goodness-of-fit test was 0.668

Regarding empiric antimicrobial treatment and outcomes for MRD ESKAPEEc BSI, 42 of the 124 MDR-ESKAPEEc patients with BSI (33.9%) were treated inappropriately in comparison with the 4 of the 44 non-MDR ESKAPEEc patients with BSI (9.1%) (*P* = 0.002) (Table 3). Hospital stay in MDR-ESKAPEEc BSI group was longer than that in non-MDR ESKAPEEc BSI group (median days, 24.0 [13.0–36.0] vs 14.5 [9.0–27.3], *P* = 0.006) (Table 3). Whereas, no significant differences were ascertained in MODS, septic shock, mechanical ventilation, PICU admission, mortality between MDR and non-MDR ESKAPEEc BSI groups (all *p* > 0.05).

### Predictors for mortality among hospitalized children with ESKAPEEc BSI

A total of 174 hospitalized children with ESKAPEEc BSI were identified in our study. The overall mortality rate of these patients was 14.4% (25/174), and no significant difference was ascertained regarding mortality between MRD-ESKAPEEc and non-MRD ESKAPEEc BSI groups (13.7% vs. 11.4%, *P* = 0.692) (Table 3). In the univariate analysis (Table 4), previous surgery and/or trauma, previous antibiotic use, neutrophil percentage, hemoglobin, platelet count, MODS, blood products transfusion, mechanical ventilation, PICU admission were statistically differences between survivor and non-survivor groups (all *p* < 0.05). A multivariate analysis demonstrated that previous surgery and/or trauma (OR = 7.006, *P* = 0.006), mechanical ventilation (OR = 7.997, *P* = 0.004) appeared to be effective predictors for death. Previous antibiotic use (OR = 0.132, *P* = 0.034), normal platelet count (OR = 0.996, *P* = 0.037) were protective factors for death caused by ESKAPEEc BSI.

**Table 4:** Analysis of predictors for mortality in 174 hospitalized children with ESKAPEEc BSI

Variable	non-survivors *n* = 25	survivors *n* = 149	Univariate analysis	Logistic Regression Analysis^#^	
			*P* Value	*P* Value	Odds Ratio (95% CI)
Male, No. (%)	10 (40.0)	61 (40.9)	0.930		
Age (m), Median (IQR)	3.6 (0.8–33.5)	3.0 (0.6–36.0)	0.909		
Presence of any underlying disease, No. (%)	20 (80.0)	91 (61.1)	0.068		
Premature and/or low birth weight, No. (%)	8 (32.0)	47 (31.5)	0.964		
Tumor diseases, No. (%)	4 (16.0)	25 (16.8)	1.000		
Aplastic anemia, No. (%)	1 (4.0)	2 (1.3)	0.909		
Congenital heart disease, No. (%)	1 (4.0)	10 (6.7)	0.943		
Lung disease, No. (%)	1 (4.0)	11 (7.4)	0.848		
Digestive disease, No. (%)	5 (20.0)	10 (6.7)	0.071		
Kidney diseas, No. (%)	1 (4.0)	3 (2.0)	1.000		
Other disease, No. (%)	4 (16.0)	7 (4.7)	0.088		
Nosocomial infection, No. (%)	5 (20.0)	51 (34.2)	0.156		
Previous surgery and/ or trauma (within 3 months), No. (%)	8 (32.0)	16 (10.7)	0.011	0.006	7.006 (1.761–27.876)
Previous hospitalization (within 1 month), No. (%)	5 (20.0)	28 (18.8)	1.000		
Previous antibiotic use (within 1 month), No. (%)	2 (8.0)	45 (30.2)	0.021	0.034	0.132 (0.020–0.860)
Likely source of infections					
Lung infection, No. (%)	5 (20.0)	26 (17.4)	0.979		
Abdominal infection, No. (%)	6 (24.0)	21 (14.1)	0.333		
Urinary infection, No. (%)	1 (4.0)	25 (16.8)	0.175		
Intracranial infection, No. (%)	0	2 (1.3)	1.000		
Skin or soft tissue infection, No. (%)	0	20 (13.4)	0.082		
Primary BSI, No. (%)	12 (48.0)	63 (42.3)	0.593		
Laboratory findings					
White blood cell count (*10^9),Median (IQR)	6.9 (3.5–11.3)	9.7 (5.3–13.9)	0.095		
Neutrophil percentage, Median (IQR)	41.0 (9.5–63.1)	58.0 (34.8–72.7)	0.019	0.072	0.979 (0.957–1.002)
Hemoglobin (g/L), Median (IQR)	97.0 (72.5–138.0)	110 (95–147.5)	0.026	0.218	0.990 (0.973–1.006)
Platelet count ((*10^9), Median (IQR)	134 (41–227)	216 (117.5–352.0)	0.002	0.037	0.996 (0.991–1.000)
C-reactive protein (mg/dL), Median (IQR)	29 (6–132)	13 (3–80.5)	0.068		
Elevated ALT, No. (%)	12 (48.0)	44 (29.5)	0.067		
Elevated AST, No. (%)	15 (60.0)	75 (50.3)	0.371		
MODS, No. (%)	7 (28.0)	8 (5.4)	0.001	0.752	1.288 (0.268–6.197)
Septic shock, No. (%)	4 (16.0)	9 (6.0)	0.180		
Blood products transfusion, No. (%)	23 (92.0)	103 (69.1)	0.018	0.581	0.597 (0.096–3.720)
Indwelling catheter, No. (%)	19 (76.0)	96 (64.4)	0.258		
Indwelling gastric tube, *N*(%)	14 (56.0)	61 (40.9)	0.159		
Central venous catheter, *N*(%)	10 (40.0)	66 (44.3)	0.689		
Urinary catheter, *N*(%)	2 (8.0)	5 (3.4)	0.587		
Mechanical ventilation, *N*(%)	13 (52.0)	31 (20.8)	0.001	0.004	7.997 (1.906–33.546)
PICU admission, No. (%)	9 (36.0)	22 (14.8)	0.022	0.137	3.065 (0.701–13.408)
Inappropriate empirical treatment, No. (%)	9 (40.9)*	37 (25.3)*	0.127		

The interpretation for the abbreviations were listed in Table 2 legend

Elevated ALT means ALT was > 40 U/L. Elevated AST means AST was > 45 U/L.

^#^ *P* value of Hosmer-Lemeshow goodness-of-fit test was 0.098

*Percentage derived from the results of non-survivors group (*n*=2) and survivors group (*n*=146) with resistance data

## Discussion

Data concerning the burden of ESKAPEEc BSI in hospitalized children was indeedy non-existent. Existing information was mainly in adult population with definite underly diseases. There were several findings identified in this study: (1) 76.3% (*n* = 174) of BSIs among hospitalized children were caused by ESKAPEEc (124 MDR-ESKAPEEc). (2) Several risk factors for BSI due to ESKAPEEc and associated with BSI caused by MRD ESKAPEEc were identified. (3) The overall mortality rate in patients with ESKAPEEc BSI was 14.4% (25/174), and no significant difference was ascertained in mortality rate between MRD-ESKAPEEc and non-MRD ESKAPEEc BSI groups (13.7% vs. 11.4%, *P* = 0.692). (4) Several predictors for mortality among hospitalized children with ESKAPEEc BSI were also summarized.

In the current study, 76.3% of BSIs were caused by ESKAPEEc, which was similar to the previous reports in Southwest China (58.7%) [12] and Rome (61.7%) [13], but higher than that in the US (27.2%) [11]. This discrepancy may be associated with geographical regions and study population. Previous studies frequently reported MDR bacteria were *Escherichia coli* and *Klebsiella pneumoniae*, most of which trended to be ESBL-producing organisms [23–25]. Furthermore, an upward trend in the proportion of ESBL-producing Enterobacteriaceae ESKAPEEc BSI was found [12]. Similarly, a high rate of MRD-ESKAPEEc BSI resulting from an ESBL-producing strain among children was also observed in this study. One common reason in many developing countries which may result in the high incidence of antibiotic resistant BSI was the overuse and misuse of antibiotics. Meanwhile, our study revealed that the most common carbapenem non-susceptibility strains were *Klebsiella pneumoniae* (6.8%,15/222), which was consistent with data reported by CHINET (5.5%) [26], but differed from the result from southwest China (12.8%) [12]. Studies have shown that carbapenem-resistant *Klebsiella pneumoniae* (CRKP) was relevant to high mortality [27, 28]. Yet the treatment regimens for CRKP BSI were limited in clinic. CRKP infections have posed an extreme antibiotic-resistant threat [28]. Thus, additional studies should concentrate on the molecular and epidemical mechanism of CRKP.

To our knowledge, there was no study using multivariate analyses to investigate risk factors for ESKAPEEc BSI. In the present study, we found that premature and/ or low birth weight, previous surgery and/or trauma and source of urinary tract infection were independent risk factors for ESKAPEEc BSI. Moreover, there was only two studies among adult population focusing on the risk factors for drug-resistant ESKAPEEc BSI in multivariate analyses [14, 15]. Gudiol C, et al. in 2014 have found that drug-resistant ESKAPEEc bacteremia were significantly associated with underlying diseases, previous antibiotic use, and source of urinary tract infection [14]. Marta B, et al. in 2013 have revealed that solid-organ transplant patients who had previous antibiotic use and septic shock were more likely to develop drug-resistant ESKAPEEc bacteremia [15]. Based on the existing information, it was likely that underlying diseases, previous invasive operation, previous antibiotic use and source of infection were the key factors for MRD-ESKAPEEc BSI. We further investigated the independent risk factor for MRD ESKAPEEc patients with BSI was nosocomial infection (OR = 3.314, *P* = 0.037). This result was not in line with those risk factors ascertained in the previous studies [14, 15]. The discrepancies may be owing to the diverse study population, diverse definition of MRD [14–17], diverse inclusion criteria [14–17]. Various studies have showed that ESKAPEEc pathogens, especially MRD-ESKAPEEc, have emerged as the predominant opportunistic organisms responsible for nosocomial infections [29–31], resulting in severe infection. Accompanying by nosocomial infection, there was urgently need for empiric treatment to cover MRD-ESKAPEEc pathogens among children with BSI. The association between bacterial resistance development and previous antibiotic use remained inconsistent. Some studies revealed that previous antibiotic exposure (especially carbapenem) was an independent risk factor for MRD pathogens infection [14, 15, 28, 32]. Conversely, other study has demonstrated no association was identified between MRD bacterial infections and previous antibiotic use [33], in accordance with our data. Thus, further association between bacterial resistance development and antibiotic use previous to infection need to be monitored closely.

To explore the possible influence of MRD pathogens for the outcome of patients with ESKAPEEc BSI, patients’ characteristics were systematically evaluated. The overall mortality rate among children with ESKAPEEc BSI was 14.4% (25/174), and no significant difference was ascertained in mortality between MRD-ESKAPEEc and non-MRD ESKAPEEc BSI patients (13.7% vs. 11.4%, *P* = 0.692), which was in accordance with previous studies [16, 17]. This finding reflected the fact that MRD-ESKAPEEc was not a risk factor associated with worse outcome, and although resistant strains were not easily to cure, might be less virulent. Moreover, previous studies have reported various predictors of poor outcome in patients with ESKAPEEc BSI, including female sex [16], lymphocyte counts < 300/mm^3^ [16], corticosteroid therapy [14] and ß-lactam monotherapy [14], septic shock [16, 17],ICU admission [14]. In our study, we found that mechanical ventilation (OR = 7.997, *P* = 0.004) was the strongest risk factor associated with mortality. Further, previous surgery and/or trauma and thrombocytopenia increased the risk rates for mortality among children with ESKAPEEc BSI. Thrombocytopenia was a well-known indicator of serious infections development [34, 35], which can be considered an early screening tool for poor outcome in children with ESKAPEEc BSI. Low platelet count resulting from bacterial infection was recorded closely and trended to indicate impaired production caused by bone marrow suppression and/or increased destruction owing to endothelial cell injury and platelet aggregation [36–38].

Though it is the first time to summarize the main characteristics of ESKAPEEc BSI among hospitalized children, several limitations exist. First, our study is a single-center study representing most of the pediatric population in the Southwest China, some findings may not be generalizable to other settings. Second, selection and recall bias occurred inevitably due to the retrospective analysis and these variations may partly affect the findings of the study.

## Conclusions

The present study revealed that more than two-thirds of BSI among hospitalized children were caused by ESKAPEEc. It also demonstrated that previous surgery and/or trauma, thrombocytopenia and mechanical ventilation increased the risk rate for mortality in ESKAPEEc BSI. The risk factors ascertained could assist physicians to early suspect ESKAPEEc BSI and MDR ESKAPEEc BSI.

## Data Availability

The datasets used during the current study are available from the corresponding author on reasonable request.

## Abbreviations

ALT: Alanine aminotransferase
AST: Aspartate aminotransferase
BSI: Bloodstream infection
CI: Confidence interval
MODS: Multiple organ dysfunction syndrome
MDR: Multidrug resistant
IQR: Interquartile range
OR: Odds ratio
PICU: Pediatric intensive care unit
